# Guidelines for the diagnosis and management of chylomicron retention disease based on a review of the literature and the experience of two centers

**DOI:** 10.1186/1750-1172-5-24

**Published:** 2010-09-29

**Authors:** Noel Peretti, Agnès Sassolas, Claude C Roy, Colette Deslandres, Mathilde Charcosset, Justine Castagnetti, Laurence Pugnet-Chardon, Philippe Moulin, Sylvie Labarge, Lise Bouthillier, Alain Lachaux, Emile Levy

**Affiliations:** 1 Department of Nutrition, CHU Sainte-Justine Research Center, Université de Montréal, 3175, Ste-Catherine Road, Montreal, Quebec, H3T 1C5, Canada; 2 Université Lyon 1; UMR 870, INSERM 8-870, INRA U-1235, Hospices Civils de Lyon, Lyon F-69002, France; 3 Department of Nutrition-Hepatogastroenterology, Hôpital Femme Mère Enfant, Bron, Université Lyon 1, Lyon F-69003, France; 4 UF Dyslipidemia Laboratory, Centre de Biologie Est, Biochemistry Laboratory, and Department of Endocrinology, Hôpital Neurologique et Cardiologique, Hospices Civils de Lyon, Lyon F-69003, France; 5 Department of Pediatrics, CHU Sainte-Justine Research Center, Université de Montréal, 3175, Ste-Catherine Road, Montreal, Quebec, H3T 1C5, Canada

## Abstract

Familial hypocholesterolemia, namely abetalipoproteinemia, hypobetalipoproteinemia and chylomicron retention disease (CRD), are rare genetic diseases that cause malnutrition, failure to thrive, growth failure and vitamin E deficiency, as well as other complications. Recently, the gene implicated in CRD was identified. The diagnosis is often delayed because symptoms are nonspecific. Treatment and follow-up remain poorly defined.

The aim of this paper is to provide guidelines for the diagnosis, treatment and follow-up of children with CRD based on a literature overview and two pediatric centers 'experience.

The diagnosis is based on a history of chronic diarrhea with fat malabsorption and abnormal lipid profile. Upper endoscopy and histology reveal fat-laden enterocytes whereas vitamin E deficiency is invariably present. Creatine kinase (CK) is usually elevated and hepatic steatosis is common. Genotyping identifies the *Sar1b *gene mutation.

Treatment should be aimed at preventing potential complications. Vomiting, diarrhea and abdominal distension improve on a low-long chain fat diet. Failure to thrive is one of the most common initial clinical findings. Neurological and ophthalmologic complications in CRD are less severe than in other types of familial hypocholesterolemia. However, the vitamin E deficiency status plays a pivotal role in preventing neurological complications. Essential fatty acid (EFA) deficiency is especially severe early in life. Recently, increased CK levels and cardiomyopathy have been described in addition to muscular manifestations. Poor mineralization and delayed bone maturation do occur. A moderate degree of macrovesicular steatosis is common, but no cases of steatohepatitis cirrhosis.

Besides a low-long chain fat diet made up uniquely of polyunsaturated fatty acids, treatment includes fat-soluble vitamin supplements and large amounts of vitamin E. Despite fat malabsorption and the absence of postprandial chylomicrons, the oral route can prevent neurological complications even though serum levels of vitamin E remain chronically low. Dietary counseling is needed not only to monitor fat intake and improve symptoms, but also to maintain sufficient caloric and EFA intake.

Despite a better understanding of the pathogenesis of CRD, the diagnosis and management of the disease remain a challenge for clinicians. The clinical guidelines proposed will helpfully lead to an earlier diagnosis and the prevention of complications.

## Background

Chylomicrons, the principal carriers of dietary lipids, are triglyceride (TG)-rich lipoproteins secreted exclusively from the enterocyte. These large lipoproteins (700 to 6000 Å) contain a single molecule of apolipoprotein (apo) B-48, which is essential for chylomicron structure cohesion [1,2]. Apo B-100 is found within very-low-density lipoproteins (VLDL) secreted by the liver and in low-density lipoprotein (LDL), a catabolic product of VLDL.

**Figure 1:**
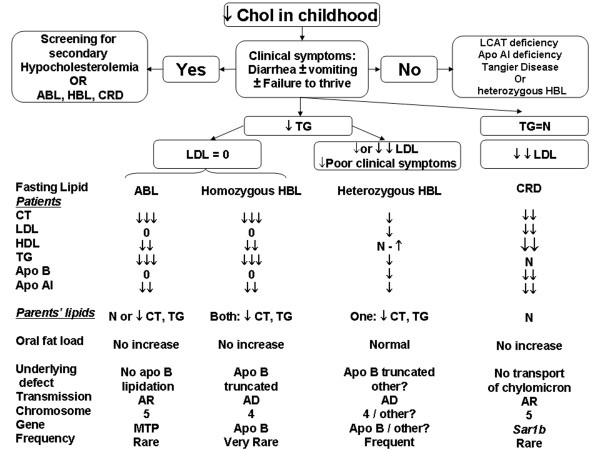
**Etiology of familial hypocholesterolemia in childhood depending on lipid profile**. ABL, abetalipoproteinemia; AD, autosomal dominant; AR, autosomal recessive; apo AI, apolipoprotein A1; apo B; apolipoprotein B; HDL, high-density lipoprotein; HBL, hypobetalipoproteinemia; LCAT, lecithin cholesterol acyltransferase; LDL, low-density lipoprotein; MTP, microsomal triglyceride transfer protein; N, normal; 0, nul; PL, phospholipids; TC, total cholesterol; CRD, chylomicron retention disease; TG, triglyceride; ↓, few decrease; ↓↓, significant decrease; ↓↓↓, intense decrease.

Many genetic diseases are responsible for alterations in apo B synthesis, metabolism or secretion abnormalities, causing intestinal fat malabsorption with growth retardation and neuro-ophtalmological complications. Over the last 20 years, genetic abnormalities have been identified for three main disorders classified as familial hypocholesterolemia: hypobetalipoproteinemia (HBL), abetalipoproteinemia (ABL) and chylomicron retention disease (CRD). Figure 1 illustrates the characteristic lipid screening profiles and oral responses to a fat load test in patients and their parents with these disorders.

HBL is due to a mutation in the *apo B *gene on chromosome 2 leading to a shorter apo B molecule (truncated apo B) [3]. The clinical phenotype of this autosomal dominant hypocholesterolemia (Figure 1) is variable, as homozygous patients are indistinguishable from ABL, while heterozygotes show only a very mild phenotype [4].

The genetic abnormality leading to ABL was identified in 1992 [5] and is due to the mutation of the microsomal triglyceride transfer protein gene on chromosome 4 [5-7]. This mutation leads to premature degradation of nascent apo B and then to a drastic decrease in apo B-containing lipoproteins.

Recently, the *SAR1B *gene was identified as responsible for CRD or Anderson's disease (MIM #607689) [8]. The *SAR1B *gene encodes the Sar1b protein, which is involved in chylomicron transport from the endoplasmic reticulum (ER) to the Golgi apparatus [9,10]. In fact, Sar1-GTP forms a coating protein complex (COPII) with two heterodimers Sec23/24 and Sec 13/31, which initiates budding and captures cargo to eject vesicles from the ER to the Golgi apparatus. When *Sar1b *is muted, the pre-chylomicron transport vesicle delivered by the ER cannot fuse with the Golgi apparatus, which then induces an accumulation of pre-chylomicron transport vesicles in the cytoplasm of the enterocytes [11-14]. Genotyping has revealed that Anderson's and CRD are in fact the same disease [8]. Recently, we described three new mutations in addition to the eight original genetic defects and a few others have been reported [8,11,15,16]. These studies have greatly contributed to a better understanding of CRD for the clinicians who often experience difficulties with the diagnosis and management of CRD patients. First, the diagnosis is often delayed because symptoms are nonspecific (diarrhea, abdominal distension, vomiting, and failure to thrive) and hypocholesterolemia may be attributed to malnutrition secondary to chronic diarrhea. Secondly, follow-up and treatment are poorly defined for many reasons. The similarity with other types of familial hypocholesterolemias may lead to a wrong diagnosis unless molecular testing is performed. Furthermore, CRD is rare: only ~ 40 cases and two small cohorts with seven and eight patients each have been described [17-29]. Very long-term follow-up into adulthood is poorly documented. However, a few authors report some adult cases with serious neurological impairment, albeit less severe than in patients with homozygous ABL or HBL [20,21,27-29]. Finally, to our knowledge, there are no specific recommendations for CRD follow-up or treatment. For the most part, therapeutic suggestions have been elaborated based on recommendations for ABL or symptomatic HBL patients. As the pathogenesis of these diseases differs, their manifestations and complications lead to poorer outcomes.

The aim of this paper is to provide an overview of the disease, comment on recent findings obtained from a cohort of 16 patients in whom a molecular diagnosis was made and for whom a median follow-up of five and ten years was available for the Montreal and Lyon cohorts, and make recommendations for the diagnosis and treatment respectively [30].

### CRD Diagnosis

CRD diagnosis can be confused with other genetic hypocholesterolemias characterized by decreased LDL-cholesterol (LDL-C), such as ABL or homozygous HBL, or with acquired disorders associated with low high-density lipoprotein-cholesterol (HDL)-C. Figure 1 summarizes the differences in familial hypocholesterolemia with low LDL-C. The major clinical and biological manifestations as well as complications are summarized in Table 1 and steps to diagnose CRD are presented in Table 2.

**Table 1:** Clinical and Biological Expressions of Chylomicron Retention Disease in Studies with Genotyping

Clinical signs	Age at onset of symptoms	Transient or permanent symptoms	Prevalence in childhood	Power of discrimination
**Anthropometry**				
Failure to thrive	Infancy (1 to 6 m)	transient if low LCFA diet	80%	+
**Gastrointestinal symptoms and signs**				
Diarrhea	Infancy (1 to 6 m)	transient if low LCFA diet	100%	+
Vomiting	Infancy (1 to 6 m)	transient if low LCFA diet	60%	+
Abdominal distension	Infancy (1 to 6 m)	transient if low LCFA diet	65%	+
Hepatomegaly, Steatosis	Infancy or late childhood	transit or permanent	15%	
**Neurology**				
Retinopathy	Adult	permanent	100%	+++
Visual abnormalities	Late childhood or adult (6 to 10 y)	transient if early treatment	30%	+
Hypo or Areflexia	Late childhood or adult (4 to 10 y)	permanent	5%	+
Myopathy	Adult	permanent	0%	+
EMG abnormalities	Late childhood or adult (4 to 10 y)	transient if early treatment	25%	+
Decreased proprioception	Late childhood or adult (4 to 10 y)	transient if early treatment	0%	+
**Cardiomyopathy/Biological signs**	Adult	permanent?	0%	+
Normal TG^§^	Infancy (1 to 6 m)	transit or permanent	90%	+++
Low Total cholesterol^§§^	Infancy (1 to 6 m)	permanent	100%	moderate decrease ++
Low LDL^†^	Infancy (1 to 6 m)	permanent	100%	moderate decrease ++
Low HDL^††^	Infancy (1 to 6 m)	permanent	100%	+++
Normal Fasting lipids in parents			100%	+
High CK (N < 100 mmol/L)	Infancy (1 to 6 m) - (460 ± 100)	permanent	60%	+++
Hepatic cytolysis (ALT < 40 mmol/L)	Infancy to late childhood - (60 ± 20)	transient or permanent	95%	+
Vitamin E deficiency (N > 18.4 μmol/L)	Infancy (1 to 6 m) - (2.7 ± 0.3)	permanent	95%	+
Vitamin A deficiency (N > 1.61 μmol/L)	Infancy (1 to 6 m) - (0.8 ± 0.5)	transit if supplementation	70%	+
Vitamin D deficiency (N > 50 nmol/L)	Infancy (1 to 6 m) - (31 ± 17)	transit if supplementation	45%	+
Vitamin K deficiency (N > 2.26 mmol/L)	Infancy (1 to 6 m) - (1.15 ± 0.6)	transit if supplementation	45%	+
Steatorrhea (N < 5 g/d)	Infancy (1 to 6 m) - (7.5 ± 3.6)	transit or permanent	85%	+
Negative Oral Fat Load	Infancy (1 to 6 m)	Permanent ?	100%	+
No acantocytosis	Infancy (1 to 6 m)	transit or permanent	90%	+++
EFA deficiency* (20:3n-9/20:4n-6)	Infancy to late childhood	permanent but variations	55%	+
**Endoscopic and histological signs**				
White duodenal mucosa	Infancy (1 to 6 m)	permanent? Fat load dependent	100%	++
Enterocyte vacuolization, chylomicron-like	Infancy (1 to 6 m)	permanent? Fat load dependent	100%	++

+: no; ++: few; +++: highly discriminative; N: normal

^§ ^X value (mmol/L) is the same in CRD as in controls (0.73)

^§§ ^X value (mmol/L) is decreased by 60% in CRD (1.49 ± 0.56) vs controls (3.73 ± 0.80)

^† ^X value (mmol/L) is decreased by 75% in CRD (0.69 ± 0.38) vs controls (2.33 ± 0.64)

^†† ^X value (mmol/L) is decreased by 5% in CRD (0.46 ± 0.08) vs controls (1.07 ± 0.22)

*X ratio in CRD is 0.04 ± 0.02 and 0.01 ± 0.005 in controls

**Table 2:** Chylomicron Retention Disease Diagnosis

**Clinical**	
**Anthropometry**	Constant but unspecific failure to thrive in early infancy (1-6 months)
**Digestive**	Chronic malabsorptive diarrhea in early infancy, frequent vomiting and abdominal distension in early infancy
**Neurology**	Areflexia, ↓ deep proprioception, and ataxia are uncommon during childhood and there is no retinopathy
**Biological **(Fasting State)	
**Lipids**	In patients with a suggestive profile:
	↓ HDL and N^al ^TG are the most discriminative specificities of CRD.
	↓↓ Total cholesterol and ↓↓ LDL: intensity of decrease only around 50% normal values
**Neuromuscular**	↑ (1.5-4N) CK discriminative but inconstant abnormalities for CRD
**Blood Cell Count**	Absence of acanthocytosis in infancy is more frequent in CRD than in AB or HB
**Hepatic**	Frequent and early but not specific ↑ (1.5-3N) AST and/or ALT, with normal GGT, bilirubin and alkaline phosphatase
**Liposoluble vitamins**	Unspecific decrease ↓↓↓ E is the most severe and only permanent vitamin deficiency even with supplementation, ↓↓ A, ↓ - N^al ^D, ↓ - N^al ^K
**Coagulation**	↓ - N^al ^INR. Decreased INR if there is vitamin K deficiency
**Plasma fatty acid**	Abnormal profile, omega 6 linoleic acid deficiency, normal omega 3
**Fasting lipids in parents**	N^al^
**Oral Fat Load**	
TGs are normal at baseline but do not increase postprandially and lack of change in low HDL after fat load	
**Upper Endoscopy **(fasting state or after enriched fat diet for 3 days)	
Unspecific white duodenal mucosa	
Optic microscopy	Villi are normal but the enterocytes are grossly distended by lipid droplets
Electron microscopy	Chylomicron-like aggregates, membrane bound?
**Genotyping**	
Mutations in *SAR1B*, Chromosome 5	
**Summary**	
1) Chronic diarrhea in young infants (< 6 Mo). Normal TG with decreased total cholesterol, LDL-C and HDL-C	
2) Failure to thrive	
3) White duodenal mucosa at endoscopy→ genetic hypocholesterolemia?	
4) Genetic mutation of SAR1B → CRD confirmed	

ALT, alanine aminotransferase; AST, aspartate aminotransferase; GGT, gamma-glutamyltranspeptidase; HDL-C, high-density lipoprotein-cholesterol; LDL-C, low-density lipoprotein-cholesterol; TG, triglycerides; INR, international normalized ratio; N^al^, Normal

### Clinical Signs

In CRD, consanguinity is frequent but there is usually no noted intrauterine growth retardation. Digestive symptoms are most prominent at the beginning of life. Nonspecific malabsorptive diarrhea is constant and begins in infants shortly after birth. Other digestive symptoms, such as vomiting or abdominal swelling, are often present. They get better within a few days or weeks with a low-fat diet. The long-term evolution of digestive symptoms is variable. The intensity of symptoms decreased over time independent of the level of fat in the diet in some previously reported patients [22]. However, our data do not support the hypothesis of intestinal adaptation during CRD. Even though our patients' gastrointestinal symptoms improved on a low-fat diet, diarrhea began again when fat was reintroduced, even in adult patients. Furthermore, there was no improvement in steatorrhea after an average of five years of follow-up [30].

Hepatomegaly is reported to occur in about 20% of CRD patients. Hepatic steatosis is a well known complication of HBL. However, in CRD, at ultrasonography hepatomegaly and steatosis were detected in a few cases. A moderate degree of macrovesicular steatosis was previously reported [22,27], but, to our knowledge, no case of cirrhosis has been reported in CRD, in contrast to ABL and HBL [31-33]. Interestingly, during follow-up, we did not find any correlation between the level of hepatic enzymes and steatosis or hepatomegaly. Serological testing alone is therefore insufficient to detect hepatic morphological problems.

Neurological, muscular or ophthalmic manifestations may raise an alarm in those older patients with a delayed diagnosis as poor compliance to treatment.

The neurological abnormalities described in CRD children are: areflexia at ten and 13 years in a French publication and 13 and 18 years in Strich's publication; areflexia combined with proprioceptive abnormalities at ten and seven years in a Canadian study [21,27,29]. Furthermore, more severe neurological degeneration, such as ataxia, myopathy and sensory neuropathy, has been reported in CRD adults 21 and 55 years old [20,28]. The mean age in the literature of children with clinical neurological manifestations is 12 years, which is significantly older than that for asymptomatic patients (four years). In our cohort, 4/16 patients had electromyographic abnormalities (electromyography with a reduction in sensory nerve conduction velocity and decreased sensory nerve action potential amplitudes). The youngest was 6.5 years old and only one had areflexia as early as age 11. Vitamin E status plays a pivotal role in neurological degenerative complications [34,35]. In our study, it is relevant to note that the patients with the more pronounced abnormalities had also the lowest vitamin E levels at diagnosis.

Muscular pain and cramps are rare, but classical complaints have been reported by patients. Recently, cardiomyopathy with a decrease in ejection fraction to 40% (normal > 60%) has been described in adults with CRD [15], but its prevalence is unknown. In our cohort, all patients had normal echocardiography, and no signs of cardiomyopathy were found before 23.5 years of age. Muscle histology did not reveal significant lipid accumulation or necrosis, but rather only mild and nonspecific abnormalities: a mild increase in lipid content, focal disorganization of the Z line with the loss of a few Z lines in specific regions of the muscle specimen (personal data), and a variation in muscle fiber diameter with numerous lobulated fibers [15].

With regard to ophthalmic complications, minimal visual abnormalities were previously been reported: micronystagmus, mild deficit in the perception of the blue-yellow axis, and delayed dark adaptation [27]. In our populations, ophthalmic complications were mild, evidenced only through functional testing (abnormal visual evoked potentials characterized by waves of higher amplitude and increased latencies, as well as abnormal scotopic electroretinograms). In conclusion, in children, the absence of severe neurologic impairment and retinopathy is compatible with a diagnosis of CRD.

Poor mineralization and delayed bone maturation have previously been observed in CRD [21], probably as a consequence of malabsorption, malnutrition and vitamin D deficiency.

Interestingly, Sar1b GTPase is expressed not only in the intestine but also in the liver, muscle and brain. The same mutation may be involved in different tissue-specific failures, for example, hepatocyte transport of nascent VLDL from the RE to the Golgi thanks to a Sar1b-dependant mechanism [36]. We can hypothesize that this dysregulation may be involved in the hepatic steatosis described in CRD. Furthermore, the myocardium has been shown to express the *Sar1b *gene and secrete apo B lipoproteins [37,38]. This suggests that the recent description of cardiomyopathy in CRD may be related to the tissue-specific expression of abnormal Sar1b protein. Finally, mild musculoskeletal and neurological abnormalities could also be related to impaired Sar1b in various systems [7,11].

### Biological Signs

Hypocholesterolemia associated with chronic diarrhea is common and nonspecific. However, CRD may be suspected with a particular lipid profile. First, the more than 50% decrease in total cholesterol (1.49 ± 0.56 in CRD vs 3.73 ± 0.80 mmol/L in control patients), LDL-C (0.69 ± 0.38 in CRD vs 2.33 ± 0.64 mmol/L in control patients) and HDL-C (0.46 ± 0.08 in CRD vs 1.07 ± 0.22 mmol/L in control patients) in the presence of normal triglycerides (TG) (0.73 mmol/L) is almost pathognomonic [30,39]. In contrast, both AB and homozygous HB have hardly any measurable TGs much lower total cholesterol levels and no LDL-C. Interestingly, in CRD, the intense hypocholesterolemia is not associated with consequences as severe as in the AB or HB homozygotes. First, the dyslipidemia is much less severe than in AB and HB. Secondly, in CRD, the chylomicrons are not secreted in the classic COP II way, but are built into the RE. Intestinal biopsies show chylomicron-like particles in membrane-bound compartments, reminiscent of the vesiculated channels of the smooth ER and in big membrane-bound compartments [19]. We can, therefore, presume that some fat is transported via LDL particles throughout the day and hardly detectable during the 5-hour fat loading test. However, at the puncture, no data support this possibility.

CRD and AB are recessive autosomal diseases, unlike HB which is a dominant form of hypocholesterolemia. Therefore, parental lipid screening may clarify the diagnosis. The absence of hypocholesterolemia in the two parents favors CRD.

Interestingly, the measurement of creatine kinase (CK) may orient towards the diagnosis. Recently and for the first time, muscular abnormalities have been described in CRD patients [15]. In our cohort, CK levels were elevated (5 × N) in all patients in whom the measurement was made at diagnosis, except one. The highest level (10 × N) was found in a patient who had the most severe level of neurological impairment (areflexia and abnormal electromyography). However, the CK level does not always correlate well with the severity of the neurological impairment. In conclusion, an elevated level of CK in a patient with hypocholesterolemia can suggest CRD.

Acanthocytes can be seen in advanced liver disease, rare vitamin E genetic deficiency or neurodegenerative syndromes (McLeod), AB, or homozygous HB. In CRD, acanthocytosis is rare, and sometimes transient. It is dependent on the vitamin E level which is a key to red cell membrane integrity. Therefore, the presence of acanthocytosis is hardly compatible with CRD.

As previously mentioned, hepatic abnormalities may be present. Nonspecific hepatic cytolysis is very frequent, but moderate, (1.5-3 × N), and poorly correlates with steatosis and/or hepatomegaly.

Malabsorption of fat leads to deficiency of fat soluble vitamins and essential fatty acid (EFA). Steatorrhea at the time of diagnosis (usually a few months after the onset of the first symptoms) is invariably present and depends on the quantity of fat ingested. With a low-fat regimen, steatorrhea may be falsely negative. The calculation of % fat absorption on a normal fat diet is a key diagnostic test in all patients suspected of CRD.

Fat malabsorption alters fat-soluble vitamins in variable ways. Vitamin E is the most affected among the liposoluble vitamins in CRD, because its transport is highly dependent on apo B-containing lipoproteins [40]. Vitamin E deficiency is constant at diagnosis in young children. In CRD, the decrease is drastic and most of the time permanent, even with vitamin E supplementation. Vitamin A is often decreased, but easily corrected with oral supplementation. Finally, vitamin D or K insufficiency detected in half the patients can also be corrected with supplements.

Fat malabsorption also affects the EFA profile. At diagnosis, our CRD patients had abnormal plasma fatty acid profiles, as evidenced by n-6 deficiency (linoleic acid; C18:2n-6) and, surprisingly, normal omega-3 levels. In addition, EFA deficiency manifested by a high 20:3n-9/20:4n-6 ratio, (0.04 ± 0.02 in CRD vs 0.01 ± 0.005 in control patients); normal value <0.02 was observed in the Canadian cohort [30].

The oral fat loading test helps evaluate intestinal fat absorption. Patients fast for 12 h and ingest 50 g of fat per 1.73 m^2 ^surface area (flavored commercial cream). Plasma lipids are measured at 2, 3 and 5 h following the fat meal. All patients with CRD do not respond to an oral fat loading test: TGs are normal or slightly decreased at baseline and do not increase at 3 and 5 h while low HDL-C stays low and no chylomicrons are identified. However, this test does not discriminate CRD from the other genetic disorders with hypocholesterolemia.

### Endoscopy and Histology

**Figure 2:**
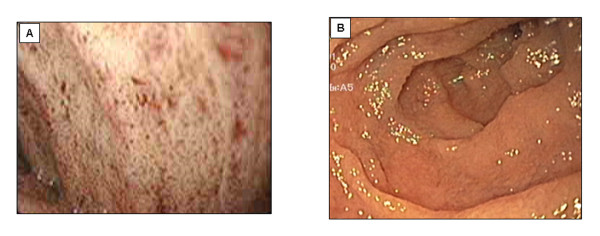
**Endoscopy of a CRD patient**. Upper endoscopy reveals a white duodenal mucosa in CRD (Panel A) compared with normal subjects (Panel B).

Upper endoscopy reveals a white duodenal mucosa with normal esophageal and normal gastric mucosa (Figure 2). To increase endoscopic sensitivity, a TG-rich diet is begun three days before the exam to improve the fat loading of enterocytes.

**Figure 3:**
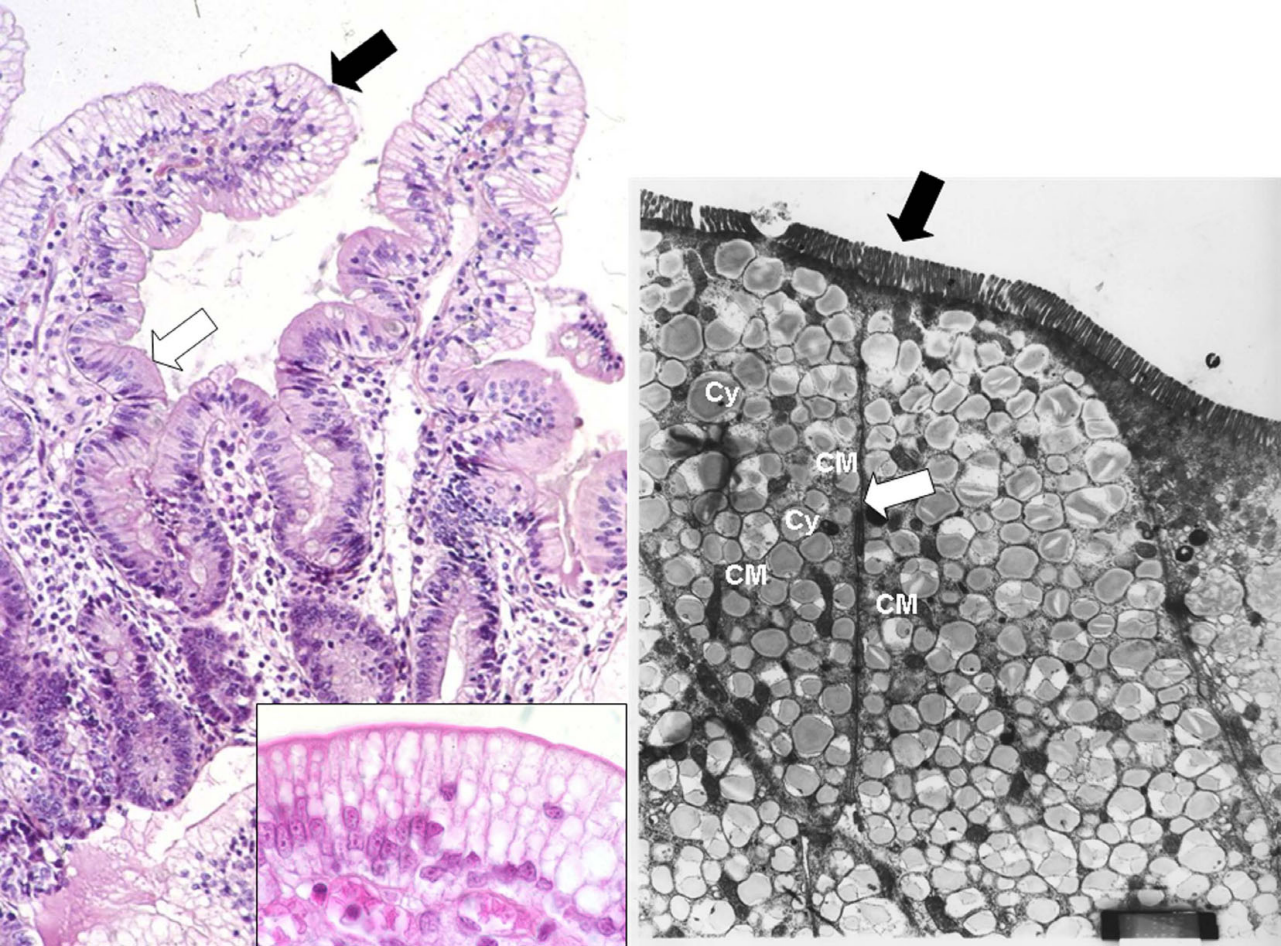
**Histology of a jejunal biopsy from a CRD patient**. Panel A: photomicrograph of hematoxylin-eosin staining showing vacuolization of enterocytes and well preserved villous structure. The distribution of vacuolization, which corresponds to lipid droplets, is heterogeneous: fat filled enterocytes (black arrow) in the upper part of the villus are associated with normal enterocytes in the crypts (white arrow) (×20). Panel B: Higher magnification (×100) of the same patient's biopsy. Panel C: Electronic microscopy. The pictures show the apical pole of the enterocytes exhibiting well-preserved microvilli (black arrow), numerous chylomicrons (CM) and fat droplets of homogenous size gorging the cytoplasm (Cy). The intercellular membranes demonstrate a complete juxtaposition of intercellular membranes where lipid particles are absent (white arrow) (×4000).

Histology shows multi-vacuolated enterocytes in intestinal villi of normal architecture (Figure 3). However, some patients demonstrate mild atrophic villi [21], which may be confused with celiac disease if lipid vacuoles are not identified. Furthermore, in the same patient, villi are affected to a variable extent and, among different patients, the percent of lipid-laden villi and the region of affected villi can vary substantially [19]. In most cases, the vacuolization is seen only in the upper one-third of the villi.

### Genetics

**Figure 4:**
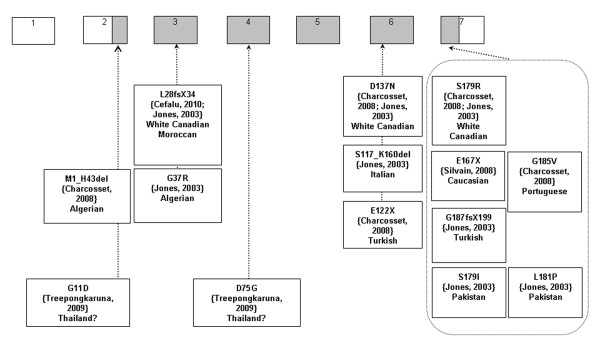
**Mutations of *SAR1B *gene as described in the literature**. Encoding exons are in grey colour. The nomenclature used is the same as that reported by Jones et al. [8] Sequences of SAR1B gene (NC_000005, gi: 51511721) and mRNA (NM_016103, gi: 38176155) are available on GenBanck (http://www.ncbi.nlm.nih.gov). Only predicted consequences of mutations are presented; mutations from 5' to 3' are: c.1-4482_58 + 1406del5946ins15pb, c.32G > A, c.83-84 delTG, c.109G > A, c.224A > G, c.349-1G > C, c.364G > T, c.409G > A, c.499G > T, c.536G > T, c.537T > A, c.542T > C, c.554G > T, c.555-558dupTTAC

Finally, identification of the *SAR1B *gene mutation confirms the diagnosis. Since the discovery of *SAR1B *as the gene that causes CRD [8], 14 different mutations in about 30 patients have been described (Figure 4) [8,11,16,41]. Only two families have had frame shift mutations that cause CRD. Until now, missense mutations have represented the majority of SAR1B mutations. We previously reported the absence of a correlation in genotype-phenotype in a cohort of 16 patients with CRD [30] and Charcosset et al. analyzed the putative consequences of different mutations including frame shift and missense mutations in CRD. Truncating variants would be expected to induce more severe phenotypes than just missense variants. However, the most deleterious mutation of lipid levels or growth reported in our cohort was a missense mutation (409G > A) [30]. Furthermore, the variation in clinical expression with the same mutation in different families [11] and even in the same family with the very surprising asymptomatic homozygote CRD mother, described by Cefalu et al [41], suggests that modifier genes may modulate the transport of the COP II vesicles and that CRD expression is more complex than a simple autosomal recessive disorder.

### CRD Management

Follow-up should be directed toward monitoring nutrition and growth, compliance to treatment and the presence of complications involving the liver, the neuromuscular system including the retina as well as bone health. Table 3 proposes a long term management program proposed for patients with CRD.

**Table 3:** Chylomicron Retention Disease Follow-Up

***Early Follow-Up *(Annual)**	
**Clinical**	
**Anthropometry**	Weight and height to draw growth curve
**Digestive**	Appetite, diarrhea, abdominal distension, vomiting, hepatic size?
**Neurological**	Developmental retardation, areflexia, ataxia, dysarthria, deep proprioception loss, muscular weakness or pain, cramps?
**Dietary counseling**	Sufficient caloric intake, low fat diet (fat <30% total energy), EFA supplementation?
**Biological**	
**Lipids**	Total and LDL cholesterol, HDL-C, TG
**Hepatic**	AST, ALT, GGT, total bilirubin, alkaline phosphatase?
**Vitamins**	Plasma levels of vitamins A, D, E and K or INR (vit K deficiency)
**Essential Fatty Acids**	Deficiency induced by low fat diet?
**Blood cell count**	Anemia?
***Delayed Follow-Up ***(every 3 years)	
1) After the age of 10 years	
**Hepatic**	Ultranosonography (steatosis, portal hypertension, yearly), Elastometry Fibroscan^®^? (further studies are needed)
**Neurological exam**	Clinical, creatine kinase, electromyography
**Ophthalmologic exam**	Fundus, color vision, visual evoked potentials, electroretinography
**Total body composition**	Bone mineral content for whole body
2) Adult age	
**Echocardiography**	Ejection fraction

ALT, alanine aminotransferase; AST, aspartate aminotransferase; CK, creatine kinase; EFA, essential fatty acids; GGT, gamma-glutamyltranspeptidase; HDL, high-density lipoprotein; LDL, low-density lipoprotein; TG, triglycerides

Growth is a pediatric-specific goal in patients with malabsorption syndromes. With early diagnosis and treatment, catch-up growth can be expected. However, seven of our patients with a delayed diagnosis did not even reach the 20^th ^percentile of their predicted growth potential of the 40^th ^percentile. Tracking the growth curve is essential in the follow-up of CRD children. Annual evaluation is appropriate.

The evolution of hepatic cytolysis seems favorable. However, the lack of very long-term data means that patients should be followed carefully. Therefore, measurement of hepatic transaminase levels and ultrasonography should both be carried out. An evaluation every three years after the age of ten seems a reasonable frequency for these noninvasive low-cost exams. Liver stiffness measured by elastometry (Fibroscan) could be a useful tool to detect early fibrosis, but this proposal needs further evaluation.

Neurological, muscular and ophthalmologic complications in CRD are less severe than in ABL or homozygous HBL, but may occur during infancy and require rigorous follow-up and compliance with vitamin treatments. The lack of data on long-term outcome in treated patients with CRD means that rigorous follow-up is required. Although high CK levels may indicate severe muscular impairment, they are inadequate to evaluate neuromuscular progression. Therefore, beginning at pre-puberty (ten years old), neurological and ophthalmologic examinations may be necessary every three years to detect complications such as developmental retardation, areflexia, ataxia, dysarthria, loss of deep proprioception, muscular weakness and decreased night vision. Furthermore, it seems reasonable to schedule a specialized neurology and ophthalmology consultation every three years to conduct electromyography, measure visual evoked potentials, and perform electroretinography, because electrical abnormalities occur before clinical symptoms. Finally, we suggest echocardiography in adulthood (> 18 years), since early detection of cardiac impairment may motivate patients who have discontinued their vitamin therapy to restart it. It may also help to schedule treatment before clinical manifestations of cardiac insufficiency develop. A frequency of every three years seems reasonable for this noninvasive exam.

Dual-energy X-ray absorptiometry (DEXA) may be performed to measure the bone mineral content of the total skeleton, the best index of bone mineralization in children [42,43].

### CRD Treatment

Table 4 summarizes our proposed treatment protocol. A low-fat diet containing an appropriate amount and ratio of n-6/n-3 is necessary to improve digestive symptoms. In very young children, milk preparations with medium-chain TG may improve diarrhea and correct malnutrition within a few days but tolerance can be a problem. In older children, a regimen low in long-chain fatty acids is usually sufficient to decrease symptoms.

**Table 4:** Chylomicron Retention Disease Treatment

Early diagnosis without neurological complications: PO	Delayed diagnosis and neurological complications: PO + IV
**Diet**	
Low-fat diet	
Enriched in essential fatty acids (vegetable oils, fish...)	
± Enriched in medium-chain triglycerides	
**Liposoluble Vitamins PO**	
Vitamin E (hydrosoluble form): 50 IU/kg/d	
Vitamin A: 15,000 IU/d (adjust according to plasma levels)	
Vitamin D: 800-1200 IU/kg/d or 100,000 IU/2 month if < 5 y old, and 600,000 IU/2 month if > 5 y old	
Vitamin K: 15 mg/week (adjust according to INR and plasma levels)	
	**One perfusion/month**
	Fatty acids: intralipid 20% 2 g/kg/month
	Vitamin E: 4 to 6 mg/kg/month
	Vitamin A: 500 IU/kg/month

PO, per os; IV, intravenous

Vitamins E and A are implicated in neurological, muscular and ophthalmic complications. High dosages of vitamin E (100 IU/kg/d) have been reported to prevent, slow or improve neurological complications in different hypocholesterolemia disorders such as ABL [35,44-48]. Furthermore, these high intakes are safe. Relatively few side effects in the short term have been reported in double-blind studies of vitamin E at doses as high as 2000 or 3200 IU/d [49]. Alpha-tocopherol either in aqueous or lipid form is the most effective form of vitamin E to prevent neurological complications. Indeed, nerve tissue is characterized by slow exchange and high selectivity for alpha-tocopherol [50]. Lipid malabsorption is a common feature of several diseases, such as celiac disease, cystic fibrosis and cholestasis. In the attempt to promote liposoluble vitamin absorption, new pharmacologic formulations of vitamin E have been developed. Tocopheryl polyethylene glycol succinate is a hydrosoluble form of vitamin E. This water-soluble formulation showed a marked and statistically significant increase in the absorption of gamma-tocopherol in malabsorbing patients with cystic fibrosis (CF) compared with a classic oil-based formulation [51]. Recently, intestinal absorption of water-soluble vitamin E (tocofersolan) was compared with a water-miscible form in 12 CF or cholestatic children [52]. Oral tocofersolan was more bio available than the water-soluble formulation in children with chronic cholestasis and similarly bio available in CF. This suggests that water-soluble vitamin E may represent an alternative to painful intramuscular vitamin E injections in chronic cholestasis, or other oral formulations in CF. However, the mechanism responsible for fat malabsorption in CRD concerns the absorptive phase as opposed to the digestive phase in CF and cholestatic syndromes. To our knowledge, no specific studies with these new vitamin E preparations have been conducted in CRD. For CRD, further investigation on the efficacy of water-soluble vitamin E is needed. Our 16 CRD patients received an average of 51 ± 32 IU/kg/d of vitamin E with some receiving a dosage as high as 100 IU/kg/d. Despite higher dosages of oral vitamins E and A and, in some cases, compliance ensured by intravenous administration, four of the seven French patients developed mild clinical signs of neurological impairment. This may be due to the delayed diagnosis or influence of the mutation. However, the majority of patients (12/16 patients) with a medium dosage of 50 IU/kg/d, sometimes as low as 20 IU/kg/d, did not develop clinical or electrical neuro-ophthalmologic complications when the treatment was given during the first years of life. Therefore, it seems reasonable to prescribe only 50 IU/kg/d of vitamin E to patients with CRD if the disease is diagnosed during the first year of life.

To prevent complications, our data do not suggest that the intravenous route is warranted. The oral route is sufficient, as illustrated by the nine patients in the Canadian cohort who were treated orally early on and who remained free of neurological complications (including two patients aged 22).

Determination of the appropriate dosage of vitamin E can be problematic. In our cohort, plasma concentration measurements were too imprecise to serve as a guide for clinicians. Plasma levels after supplementation reached only one half to two thirds of normal levels, independent of the vitamin E dose. Among the 16 patients with 20, 40 or 100 IU/kg/d, one may find the same plasma vitamin E levels of around 60% of the lower normal range. This level is comparable with that obtained in ABL after oral supplementation with high doses (45% of the normal range for 100 IU/kg/d of vitamin E) [53]. Furthermore, plasma levels of vitamin E did not necessarily correlate with vitamin E dosages. The severity of steatorrhea was not correlated with vitamin E and A deficiency in our patients. Therefore, vitamin treatment cannot be adjusted to the severity of fat malabsorption. An interesting alternative could be to measure vitamin E levels in subcutaneous adipose tissue. It was previously demonstrated that after supplementation, adipose tissue concentrations reach normal levels, even if plasma levels remain low [54].

Experience with our 16 patients suggests that vitamin A at a dosage of 15000 IU/d is effective in combination with vitamin E to prevent ophthalmologic complications in CRD.

When vitamin D treatment (800 to 1200 IU/d) is started early, it prevents osteopenia, as illustrated by the nine Canadian patients.

Vitamin K was administered at the same dosage of 15 mg/week in the two groups. The plasma values were not measured, but coagulation screening was normal and no hemorrhages were detected in any patient during the entire follow-up.

Patients with lipid malabsorption are at an increased risk for EFA deficiency. Such as mentioned previously, a low-fat diet is necessary to improve digestive symptoms, but it may increase the risk of EFA deficiency. Therefore, dietary counseling is needed not only to decrease fat intake but also to maintain sufficient caloric and EFA intake. It was recommended that the nine Canadian patients add one to three teaspoons per day of soybean oil to meals and increase fish consumption to two to three times per week. The objective was to reach 3-5% of total calories with omega 6, and 0.5-1% with omega 3. The French cohort (seven patients) had the same dietary recommendations, but each month four of them also received an infusion of 80 g of lipid emulsion containing 8 g of EFA (linoleic, 80% olive oil and 20% purified soy oil). The other three patients drank 25 g per day of oral supplements containing medium-chain TG (Liprocil, 80% MCT, Nestlé, France). Intravenous and oral supplementations were unable to normalize the EFA plasma levels. Furthermore, the clinical impact of this supplementation is not obvious, since the Canadian cohort had less supplementation and worse plasma levels, but fewer complications. This discrepancy may be explained by a difference between plasma fatty acid levels and the fatty acid concentration in tissues, since dietary fatty acids are mainly carried by chylomicron vesicles, which are dramatically decreased in CRD. Adipose tissue biopsies could provide some answers to this question. Simple counseling to increase oral EFAs with vegetable oils and fish seems appropriate in CRD, as illustrated by the excellent clinical progression of the nine Canadian patients despite the low plasma omega-6 levels. However, prospective clinical studies with fatty acid supplementation are needed to evaluate the optimal dose, route of administration and long-term impact of EFA treatment. Similarly, the role of medium-chain TG supplementation in CRD patients remains to be defined.

## Conclusions

CRD diagnosis and management remain challenging for clinicians. Significant progress has been made with regard to the pathogenesis of CRD, the relationship between genotype and phenotype and its outcome. Thanks to the experience of two medical centers and a review of the literature, this paper proposes clinical guidelines for the diagnosis, follow-up and treatment of CRD.

The major clinical and biological features of CRD are summarized in Table 2:

In infants under six months the disease presents as chronic malabsorptive diarrhea with malnutrition associated with an altered lipid profile: TGs are normal but both LDL-C and HDL-C are below 50% of normal levels, vitamin E deficiency and elevated CK are associated. In older children: there is in variably a history of chronic diarrhea with stunting of growth and delayed puberty eventually associated with mild neurological impairment and elevated CK. Hypocholesterolemia is also present with the same pattern, but may be overlooked. Chronic mild cytolysis of liver cells with an echographic pattern of steatosis may be an indication for screening. In both situations, endoscopy reveals a white duodenal mucosa by fat-laden enterocytes.

For follow-up (Table 3): During childhood, standard clinical examination and biological evaluations should be performed annually, focusing on nutrition growth, gastrointestinal, liver and neurological manifestations and complications.

In children over the age of ten, neurology and ophthalmology consultations bone densitometry should be obtained every three years. An echocardiogram can be added to those when adulthood is reached.

## Abbreviations

Å: ångström; ABL: abetalipoproteinemia; Apo: apolipoprotein; COPII: coating protein complex type II; CK: creatine kinase; CRD: chylomicron retention disease; EFAD: essential fatty acid deficiency; EFA: essential fatty acid; HBL: hypobetalipoproteinemia; HDL: high-density lipoprotein; LDL: low-density lipoprotein; TG: triglycerides; VLDL: very-low density lipoprotein.

## Competing interests

The authors declare that they have no competing interests.

## Authors' contributions

NP, AS, CCR, AL, EL: conception, acquisition/analysis of the data, manuscript writing; CD, MC, AL: conception and acquisition/analysis of the data; JC, LPC, PM, SL: analysis of the data and manuscript revision. All the authors have read and approved the final manuscript.
